# Impact of suppression of the SOS response on protein expression in clinical isolates of *Escherichia coli* under antimicrobial pressure of ciprofloxacin

**DOI:** 10.3389/fmicb.2024.1379534

**Published:** 2024-04-10

**Authors:** Esther Recacha, Benno Kuropka, Sara Díaz-Díaz, Andrea García-Montaner, Enrique González-Tortuero, Fernando Docobo-Pérez, Alexandro Rodríguez-Rojas, Jose Manuel Rodríguez-Martínez

**Affiliations:** ^1^Unidad Clínica de Enfermedades Infecciosas y Microbiología, Hospital Universitario Virgen Macarena, Seville, Spain; ^2^Centro de Investigación Biomédica en Red en Enfermedades Infecciosas (CIBERINFEC), Instituto de Salud Carlos III, Madrid, Spain; ^3^Instituto de Biomedicina de Sevilla IBIS, Hospital Universitario Virgen Macarena/CSIC/Universidad de Sevilla, Seville, Spain; ^4^Institute of Chemistry and Biochemistry, Freie Universität Berlin, Berlin, Germany; ^5^Departamento de Microbiología, Facultad de Medicina, Universidad de Sevilla, Seville, Spain; ^6^School of Science, Engineering, and Environment, University of Salford, Salford, United Kingdom; ^7^Division for Small Animal Internal Medicine, Department for Small Animals, University of Veterinary Medicine Vienna, Vienna, Austria

**Keywords:** SOS response, *Escherichia coli*, proteome profile, antimicrobial resistance, Enterobacteriaceae

## Abstract

**Introduction/objective:**

Suppression of the SOS response in combination with drugs damaging DNA has been proposed as a potential target to tackle antimicrobial resistance. The SOS response is the pathway used to repair bacterial DNA damage induced by antimicrobials such as quinolones. The extent of *lexA*-regulated protein expression and other associated systems under pressure of agents that damage bacterial DNA in clinical isolates remains unclear. The aim of this study was to assess the impact of this strategy consisting on suppression of the SOS response in combination with quinolones on the proteome profile of *Escherichia coli* clinical strains.

**Materials and methods:**

Five clinical isolates of *E. coli* carrying different chromosomally- and/or plasmid-mediated quinolone resistance mechanisms with different phenotypes were selected, with *E. coli* ATCC 25922 as control strain. In addition, from each clinical isolate and control, a second strain was created, in which the SOS response was suppressed by deletion of the *recA* gene. Bacterial inocula from all 12 strains were then exposed to 1xMIC ciprofloxacin treatment (relative to the wild-type phenotype for each isogenic pair) for 1 h. Cell pellets were collected, and proteins were digested into peptides using trypsin. Protein identification and label-free quantification were done by liquid chromatography-mass spectrometry (LC–MS) in order to identify proteins that were differentially expressed upon deletion of *recA* in each strain. Data analysis and statistical analysis were performed using the MaxQuant and Perseus software.

**Results:**

The proteins with the lowest expression levels were: RecA (as control), AphA, CysP, DinG, DinI, GarL, PriS, PsuG, PsuK, RpsQ, UgpB and YebG; those with the highest expression levels were: Hpf, IbpB, TufB and RpmH. Most of these expression alterations were strain-dependent and involved DNA repair processes and nucleotide, protein and carbohydrate metabolism, and transport. In isolates with suppressed SOS response, the number of underexpressed proteins was higher than overexpressed proteins.

**Conclusion:**

High genomic and proteomic variability was observed among clinical isolates and was not associated with a specific resistant phenotype. This study provides an interesting approach to identify new potential targets to combat antimicrobial resistance.

## Introduction

The SOS response is a conserved bacterial stress response triggered primarily by agents causing DNA damage, which includes specific antimicrobials such as fluoroquinolones. The SOS response is induced by activation of RecA protein, which binds to single-stranded DNA (ssDNA) fragments and triggers autoproteolysis of the SOS repressor, LexA, leading to the expression of genes under its control. Suppression of the SOS response by targeting RecA has been proposed as a promising strategic target to tackle antimicrobial resistance due to the multifunctional role of RecA protein involvement in DNA repair, recombination, induction of the SOS response, mutagenesis pathways, horizontal gene transfer, motility, and biofilm formation (Baharoglu and Mazel, 2014).

Previous studies, using *in vitro* and *in vivo* models, have demonstrated suppression of the SOS response as a strategy for sensitization and reversal of resistance to fluoroquinolones in laboratory strains and clinical isolates [including susceptible, low-level quinolone resistance (LLQR) and resistance phenotypes; Recacha et al., 2017, 2019; Machuca et al., 2021]. RecA inactivation resulted in up to 16-fold reductions in fluoroquinolone MICs and changes of clinical category, even in isolates belonging to the high-risk clone ST131 (Pitout and DeVinney, 2017), as well as a marked decrease in the development of resistance to these antimicrobials. These data provide further support for RecA inactivation as a promising strategy for increasing the efficacy of fluoroquinolones against susceptible and resistant clinical isolates, including high-risk clone isolates (Woodford et al., 2011; Mathers et al., 2015).

In addition, our group has shown that LLQR phenotypes significantly altered gene expression patterns in systems critical to bacterial survival and mutant development at clinically relevant concentrations of ciprofloxacin. Multiple genes involved in ROS modulation (the TCA cycle, aerobic respiration, and detoxification systems) were upregulated in LLQR mutants, and components of the SOS system were downregulated (Machuca et al., 2017).

Further studies are needed to comprehensively address the cellular and metabolic changes associated with bacterial sensitization when the SOS response is suppressed. It is also crucial to determine the extent of underexpression or overexpression of proteins from various pathways during treatment with inhibitory concentrations of fluoroquinolones such as ciprofloxacin using clinical isolates, which will provide a deeper understanding of the underlying mechanisms of resistance and tolerance. To address this question, we used a large-scale proteomics approach to determine the relative protein expression levels using a set of well-characterized clinical isolates with different levels of resistance to ciprofloxacin (from susceptible to high levels of resistance). All isolates were compared with their isogenic mutants in which the SOS response was suppressed by disrupting *recA* gene expression (Machuca et al., 2021).

## Materials and methods

### Strains, growth conditions, and antimicrobial agents

*Escherichia coli* ATCC 25922 was the bacterial model for all experiments. Five *E. coli* clinical isolates, including two belonging to the high-risk clone ST131, harboring different combinations of chromosomally- and/or plasmid-mediated quinolone resistance mechanisms with susceptible, LLQR and resistance phenotypes were selected (Table 1; Briales et al., 2012; López-Cerero et al., 2013; Rodríguez-Martínez et al., 2016; Machuca et al., 2021). The SOS response was suppressed by *recA* gene knockout resistant to kanamycin, using a modified version of the method described by Datsenko and Wanner (2000) and Machuca et al. (2021). Liquid or solid lysogeny broth (LB; Invitrogen™, Madrid, Spain) medium and Mueller–Hinton broth (MHB; Oxoid, Madrid, Spain) were used. Strains were grown at 37°C. The antimicrobials used for the various assays were ciprofloxacin and kanamycin (Sigma–Aldrich, Madrid, Spain).

**Table 1:** Genotype and susceptibility to ciprofloxacin of clinical isolates and their ∆*recA* mutants.

	Quinolone resistance genotype^a^										
Strain	*gyrA1*	*gyrA2*	*parC1*	*parC2*	PMQR	ST^b^	SOS response	CIPROFLOXACIN MIC^c^	EUCAST clinical category	Fold change CIPROFLOXACIN^d^	Source of reference
ATCC	-	-	-	-	-	ST73	WT	0.004	S		
ATCC ∆*recA*	-	-	-	-	-	ST73	∆*recA*	0.001	S	4	Recacha et al. (2017)
FI 4	-	-	-	-	*qnrB*	ST73	WT	0.5	**ATU**		
FI 4 ∆*recA*	-	-	-	-	*qnrB*	ST73	∆*recA*	0.06	**S**	8	Machuca et al. (2021)
FI 10	-	-	-	-	*qnrB*	ST93	WT	0.25	S		
FI 10 ∆*recA*	-	-	-	-	*qnrB*	ST93	∆*recA*	0.016	S	15	Machuca et al. (2021)
FI 19	S83L	D87N	S80I	-	*qnrS*	ST1421	WT	4	R		
FI 19 ∆*recA*	S83L	D87N	S80I	-	*qnrS*	ST1421	∆*recA*	2	R	2	Machuca et al. (2021)
FI 20	S83L	-	S80R	-	-	ST131	WT	0.5	**ATU**		
FI 20 ∆*recA*	S83L	-	S80R	-	-	ST131	∆*recA*	0.125	**S**	4	Machuca et al. (2021)
FI 24	S83L	D87N	S80I	E84V	-	ST131	WT	32	R		
FI 24 ∆*recA*	S83L	D87N	S80I	E84V	-	ST131	∆*recA*	4	R	8	Machuca et al. (2021)

^a^Mechanisms of quinolone resistance. Resistance-associated mutations located in the GyrA and ParC proteins are defined as resistance mechanisms that alter the target site. PMQR, Plasmid-mediated quinolone resistance genes. ^b^Sequence-type according to the MLST scheme of the University of Warwick (https://enterobase.warwick.ac.uk/). ^c^MIC (mg/L) of agents by microdilution broth. ^d^Fold reduction in MIC compared with the MIC of clinical isolates (wild-type SOS response). ATU, Area of Technical Uncertainty. S, susceptible. R, resistant.

### Ciprofloxacin susceptibility testing

The susceptibility of each bacterial strain was determined in triplicate for each bacterial strain using the broth microdilution method, according to EUCAST guidelines.^1^ Briefly, an inoculum of 5 × 10^5^ CFU/mL of bacteria diluted in Mueller Hinton-Broth was exposed to twofold dilutions of ciprofloxacin. EUCAST 2023 (v13.0) clinical breakpoints were used for interpretation. Any change in MIC of at least two dilutions was considered significant. Clinical categories were established according to EUCAST breakpoints (Table 1; Machuca et al., 2021).

### Whole genome sequencing

Whole-genome sequencing was performed to analyze the genomes of the 5 clinical isolates selected (FI 4, FI 10, FI 19, FI 20, and FI 24). Genomic DNA was extracted and sequenced on the MiSeq platform (Illumina, San Diego, CA, United States), and the library was prepared using the Nextera XT DNA library preparation kit (Illumina). Raw reads were quality filtered and assembled into contigs with CLC Genomics Workbench v.10.0 (Qiagen, Madrid, Spain). The average coverage was 50x. The resulting contigs were annotated with Prokka v. 1.14.5 (Seemann, 2014) using known proteins of *E. coli* ATCC 25922 from the UniProt release 2020_02 (The UniProt Consortium, 2018) as a reference database (“*-proteins”*), without removing the original annotation in case of conserved hypothetical proteins (“*-rawproduct”*), formatted according to NCBI standards (“*-compliant -addgenes*”) and annotating ncRNA elements using Rfam v. 14.1 (Kalvari et al., 2021; “*-rfam”*). ResFinder v.4.1 (Camacho et al., 2009; Zankari et al., 2017; Bortolaia et al., 2020) and MLST v.2. tools (Lemee et al., 2004; Bartual et al., 2005; Griffiths et al., 2010; Larsen et al., 2012)^2^ were used to identify acquired resistance genes [using an identity threshold of 90% (Zankari et al., 2012)] and determine the sequence type of each isolate, respectively. The genome assemblies were analyzed with OrthoFinder v.2.5.2 software (Emms and Kelly, 2019) to classify gene sequences into conserved gene families. Proteomic data obtained from the annotated genomes of the clinical isolates and reference strain were used as input data for OrthoFinder to find all clusters of orthologous groups (“*-M msa -oa*”; Emms and Kelly, 2015, 2019). All strain sequences were deposited in a public repository under accession number PRJNA1015411.

### Proteomics sample preparation

*Escherichia coli* strain ATCC 25922 and clinical isolates were grown at 37°C in LB medium to an OD600 of 0.5 (exponential phase). Cultures were diluted 10-fold in fresh LB. Ciprofloxacin was added to tubes at a final concentration of 1xMIC (relative to the wild-type MIC for each isogenic pair; Table 1). This concentration was sufficient to induce the relevant stress conditions without high lethality (Machuca et al., 2021) and allowed us at the same time to compare the cellular response to ciprofloxacin at identical absolute concentrations for each isogenic pair. All tubes were incubated at 37°C for 1 h with shaking. The remaining ciprofloxacin was removed by centrifugation at 10,000 × g for 2 min. After removal of the supernatant, an equivalent amount of fresh LB was added. Each experimental condition consisted of six independent replicates. One milliliter per sample was pelleted by centrifugation at 10.000 x g for 2 min. Cells were resuspended in 50 μL of TE (10 mM Tris–HCl pH 8.0, 1 mM EDTA) containing chicken lysozyme (0.1 mg/mL, Sigma Aldrich, Germany) and incubated for 5 min at room temperature with occasional swirling. A 250 μL volume of denaturation buffer (6 M urea/2 M thiourea in 10 mM HEPES pH 8.0) was added to each sample, and 25 μL (corresponding to approximately 50 μg of total protein) of the resulting lysate was used for in-solution protein digestion, as described previously (Rappsilber et al., 2007). Briefly, the proteins were re-suspended in denaturation buffer and reduced by the addition of 1 μL of 10 mM DTT dissolved in 50 mM ammonium bicarbonate (ABC) and incubated for 30 min, followed by a 20-min alkylation reaction in the dark by the addition of 1 μL of iodoacetamide at a stock concentration of 55 mM. As a first digestion step, 0.5 μg of Lysyl endopeptidase (LysC, Wako, Japan), resuspended in 50 mM ABC, was added and incubated for 3 h. After pre-digestion with LysC, the protein samples were diluted by a factor of 4 with 50 mM ABC (to reduce the concentration of urea) and subjected to overnight trypsin digestion at room temperature using 1 μg of sequencing grade modified trypsin (Promega, United States), also diluted in 50 mM ABC. The digestion was stopped by acidification by adding 5% acetonitrile and 0.3% trifluoroacetic acid (final concentrations). Samples were micro-purified and concentrated using the Stage-tip protocol, described elsewhere (Rappsilber et al., 2007), and eluates were dried under vacuum.

### Nano liquid chromatography-mass spectrometry

Peptides were reconstituted in 20 μL of 0.05% trifluoroacetic acid (TFA), 4% acetonitrile, and 5 μL were analyzed by an Ultimate 3,000 reversed-phase capillary nano liquid chromatography system connected to a Q Exactive HF mass spectrometer (Thermo Fisher Scientific). Samples were injected and concentrated on a trap column (PepMap100 C18, 3 μm, 100 Å, 75 μm i.d. × 2 cm, Thermo Fisher Scientific) equilibrated with 0.05% TFA in water. After switching the trap column inline, LC separations were performed on a capillary column (Acclaim PepMap100 C18, 2 μm, 100 Å, 75 μm i.d. × 25 cm, Thermo Fisher Scientific) at an eluent flow rate of 300 nL/min. Mobile phase A contained 0.1% formic acid in water, and mobile phase B contained 0.1% formic acid in 80% acetonitrile/20% water. The column was pre-equilibrated with 5% mobile phase B followed by an increase of 5%–44% mobile phase B in 100 min. Mass spectra were acquired in a data-dependent mode using a single MS survey scan (m/z 350–1,650) with a resolution of 60,000, and MS/MS scans of the 15 most intense precursor ions with a resolution of 15,000. The dynamic exclusion time was set to 20 s and automatic gain control was set to 3 × 10^6^ and 1 × 10^5^ for MS and MS/MS scans, respectively.

### Data analysis

MS and MS/MS raw data were analyzed using the MaxQuant software package (version 2.0.3.0) with implemented Andromeda peptide search engine (Tyanova et al., 2016a). Data of the samples from strain ATCC 25922 were searched against the *E. coli* reference proteome downloaded from Uniprot (4,857 proteins, taxonomy 83,333, last modified 1 December 2019), while data of the samples from the 5 clinical isolates (FI 4, FI 10, FI 19, FI 20, and FI 24) were searched against individual databases generated from whole-genome sequencing as described above. The default parameters were used for MaxQuant except for enabling the options label-free quantification (LFQ) and match between runs. Filtering and statistical analysis was carried out for each strain individually using the software Perseus version 1.6.14 (Tyanova et al., 2016b). First, contaminants, reverse hits and ‘proteins only identified by site’ were removed from the dataset and protein LFQ intensities were log2 transformed. Next, two experimental groups (wild-type and *recA* mutant) were defined. Only proteins which were identified and quantified with LFQ intensity values in at least 3 (out of 6) experimental replicates (in at least 1/2 experimental groups) were included for downstream analysis. Missing protein intensity values were replaced from normal distribution (imputation) using the default settings in Perseus (width 0.3, down shift 1.8). Mean log_2_ fold protein LFQ intensity differences between experimental groups (*recA* mutant—wild-type) were calculated in Perseus using a student’s *t*-test with permutation-based FDR of 0.05 to generate the adjusted *p*-values (=*q*-values). Proteins with a minimum 2-fold change in their relative intensity (log2-fold change > 1 for recA or log2-fold change < −1 for wild-type) and a q-value < 0.05 were considered significantly changed. Heatmaps and volcano plots were used to represent the results, using GraphPad Prism 8 software (Boston, Massachusetts United States).^3^

The mass spectrometry proteomics data have been deposited to the ProteomeXchange Consortium via the PRIDE partner repository (Perez-Riverol et al., 2022)^4^ with the dataset identifier PXD050358.

## Results

### Validation of ciprofloxacin susceptibility profiles

The phenotypic and genetic characteristics of the isolates are shown in Table 1 (Machuca et al., 2021).

### Analysis of genomic profile of clinical isolates

In total, 5,569 genes were found among the five selected clinical isolates and reference strain ATCC 25922: 3376 of these were present in all genomes, and 3,311 genes in a single copy. The total number of genes encoded by each isolate was 4,834, 4,811, 4,683, 4,701, 4,853, and 4,842 for ATCC, FI 4, FI 10, FI 19, FI 20, and FI 24, respectively. Between each isolate and ATCC 25922, the total number of genes in common was 4,482 (93.2%), 3,705 (79.1%), 3,647 (77.6%), 4,035 (83.1%), 4,043 (83.5%) for FI 4, FI 10, FI 19, FI 20, and FI 24, respectively.

Known and potential SOS-regulated genes described previously by Fernández De Henestrosa et al. (2000) and Courcelle et al. (2001) were found in the genomes of the selected isolates. Twenty-six of the 32 genes known to be LexA-regulated genes were present in all isolates, including ATCC 25922: *umuC, umuD, sbmC, recN, urvB, dinI, recA, sulA, uvrA, uvrB, ssb, yebG, lexA, dinF, ydjQ, ruvA, ruvB*, *molR, uvrD, dinG, yigN, ydjM, ftsK, dinB, ybfE, polB.* In addition, six of the 20 genes were identified as potential LexA-regulated genes: *ymgF, ydeO, yoaA, yoaB, glvB, ibpA* (Courcelle et al., 2001). Consequently, most of the SOS-regulated genes in *E. coli* were represented in our collection.

### Analysis of proteomic profile after treatment with ciprofloxacin

The protein expression of *E. coli* clinical isolates was compared with their isogenic pairs with suppressed SOS response under ciprofloxacin treatment at concentrations of 1xMIC relative to wild-type for 1 h.

**Figure 1:**
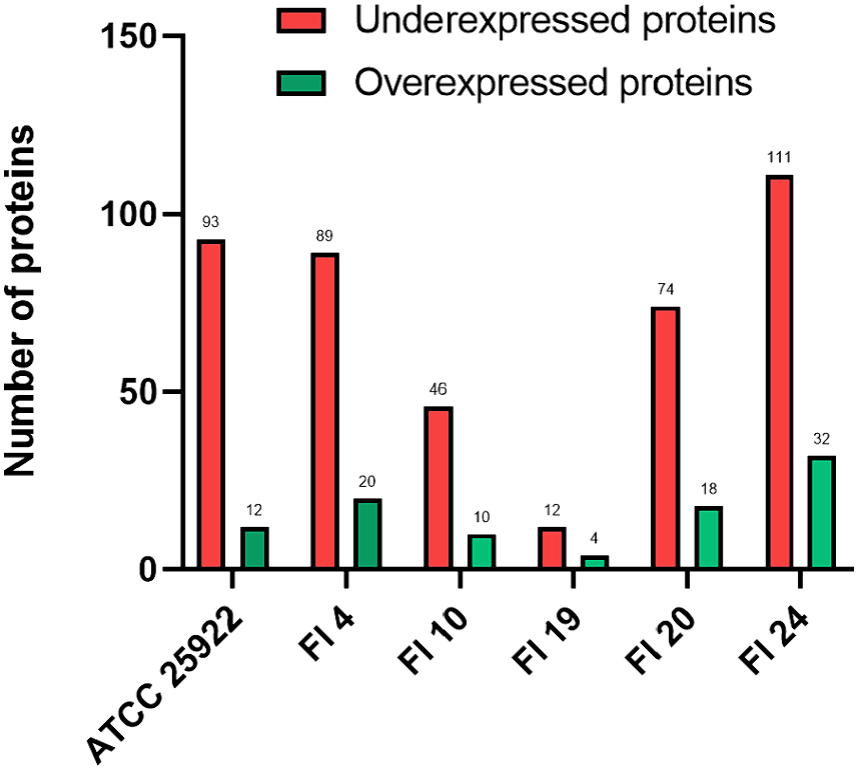
Number of significantly over- and underexpressed proteins upon deletion of *recA* for ATCC 25922 and the five clinical isolates, with at least a 2-fold change in their relative abundance (log2 fold change >1 for increased and <−1 for decreased). Proteins were considered significantly changed with an adjusted *p*-value (=*q*-value) < 0.05. All experiments were done using 1xMIC concentration of ciprofloxacin.

**Figure 2:**
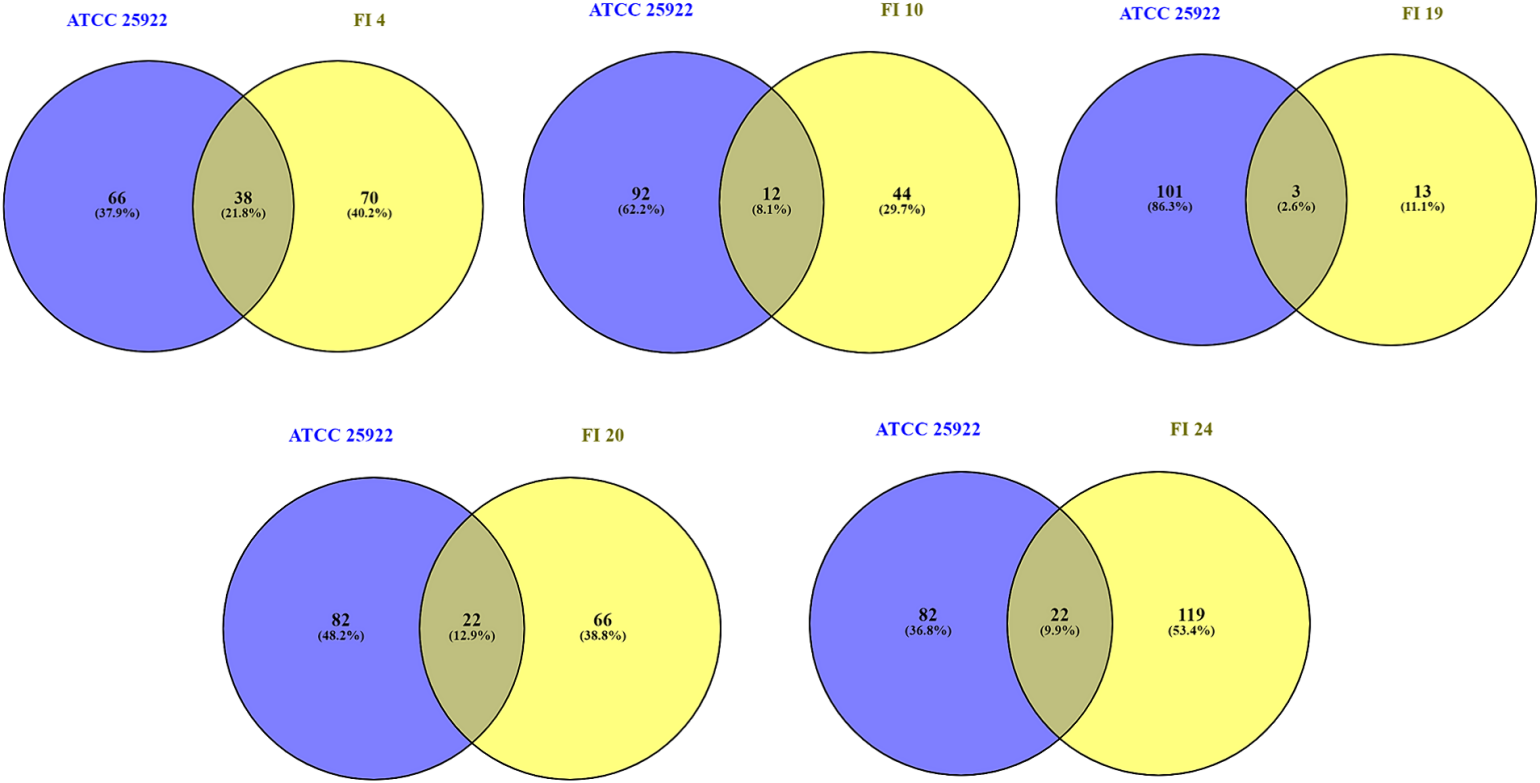
Overlap between differentially expressed proteins (log2 fold change >1 and <−1, *p*-value < 0.05) following exposure to ciprofloxacin (1xMIC relative to each wild-type) between isolates with suppressed SOS response relative to wild-type and the control strain in the same conditions. Venn diagrams show the overlap. Numbers on the diagram refer to the number of proteins with significantly altered expression levels. Susceptible phenotype: FI 10; LLQR phenotypes: FI 4 and FI 20; Resistant phenotype: FI 19 and FI 24.

Significant changes in protein expression were found for 460/1,702 proteins (27%) in ATCC, 664/1,509 (44%) in FI 4, 831/1,637 (51%) in FI 10, 904/1,726 (52%) in FI 19, 1,210/1,786 (68%) in FI 20, and 1,227/1,706 (72%) in FI 24 (see Supplementary Tables 1–6). The number of proteins that exhibited at least a significant 2-fold increase or decrease of their relative abundance upon *recA* deletion are highlighted in Figure 1. The number of proteins decreased upon *recA* deletion are: 93 for ATCC, 89 for FI 4, 46 for FI 10, 12 for FI 19, 74 for FI 20, and 111 for FI 24. The number of proteins increased upon *recA* deletion are: 12 for ATCC, 20 for FI 4, 10 for FI 10, 4 for FI 19, 18 for FI 20, and 32 for FI 24. Proteins with significant expression changes were plotted for each isolate and compared to the ATCC 25922 control strain (Figure 2), showing a similarity ranging between 4 and 22%. Regarding significant protein expression after suppression of the SOS system, 10 (DinG, DinI, RecA, RecN, RuvA, RuvB, SbmC, UmuD, UvrA, YebG) out of 32 proteins known whose genes are regulated by LexA were underexpressed in at least one isolate, and no potential protein regulated by LexA was affected.

**Figure 3:**
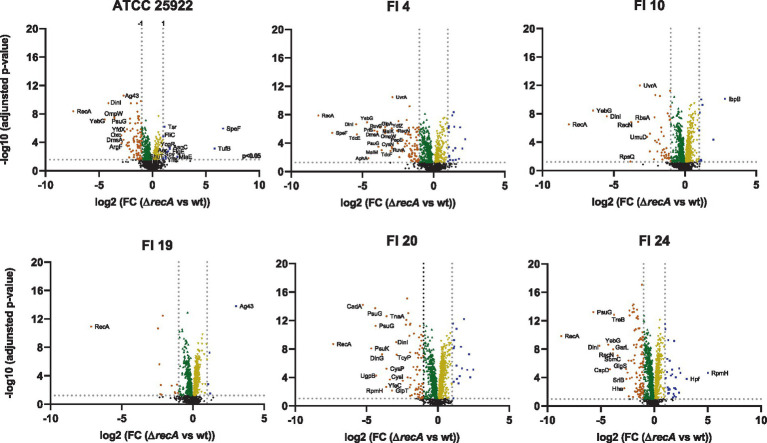
Proteome profile by strain. Orange: Proteins with log2 fold change (FC) < −1; Blue: Proteins with a log2 FC > 1; Green: Proteins with log2 FC = 0 to −1; Yellow: Proteins with log2 FC = 0 to 1. Black: no significant proteins (*p* > 0.05). Labeled proteins with log2 FC between >2.5 and < −2.5. wt, wild-type.

The relative protein intensity between the *recA* mutant and wild-type are shown in Figure 3 for ATCC 25922 and the five clinical isolates and proteins that exhibit a very strong change in their abundance upon *recA* deletion are labeled. Underexpressed proteins (log2 FC < −2.5) were: RecA (as control), YebG, DinI, OmpW, PsuG, Oxc, ArgF, Ag43, DmsA, YfdX (for strain ATCC); RecA, SpeF, DinI, TdcE, YebG, AphA, PriS, DmsA, PsuG, RuvB, MalM, MalK, OmpW, GlpA, TdcF, CysN, YdfZ, RuvA, UvrA, RecN, PepE (strain FI 4); RecA, YebG, DinI, RpsQ, RecN, RbsA, UvrA, UmuD (for strain FI 10); RecA (strain FI 19); RecA, CadA, PsuK, PsuG, UgpB, RpmH, DinG, YfeC, CysP, TnaA, CysI, GlpT, DinI, TcyP (strain FI 20) and RecA, PsuG, DinI, YebG, CspD, RecN, GarL, TreB, SbmC, Hha, GlgS, SrlB (strain FI 24). Overexpressed proteins (log2 FC > 2.5) were: SpeF, TufB (for the ATCC strain); IbpB (strain FI 10); Ag43 (strain FI 19), RpmH, Hpf (strain FI 24; Figure 3).

Protein expression under ciprofloxacin pressure was highly variable both among isolates and between isolates and the reference strain. No relationship was observed between protein expression and the quinolone resistance phenotype displayed by the different isolates. At the ciprofloxacin concentration used, underexpressed proteins were more numerous than overexpressed ones, and were mainly associated with processes of DNA repair (RecA, YebG, DinI, DinG, RuvA, RuvB, UvrA, RecN, UmuD, CspD, SbmC) and energy production and conversion (DmsA, TdcE, AphA, TdcF, CysI, CysN, CysP, TnaA). Overexpressed proteins were few and were involved in different cellular processes, such as amino acid metabolism (SpeF), translation (TufB, RpmH), protein refolding (IbpB) and biofilm formation (Ag43). Taken together, these results indicate that the treatment had a serious impact on cellular physiology.

### Functional analysis of the impact of the SOS suppression at the proteomic level

**Figure 4:**
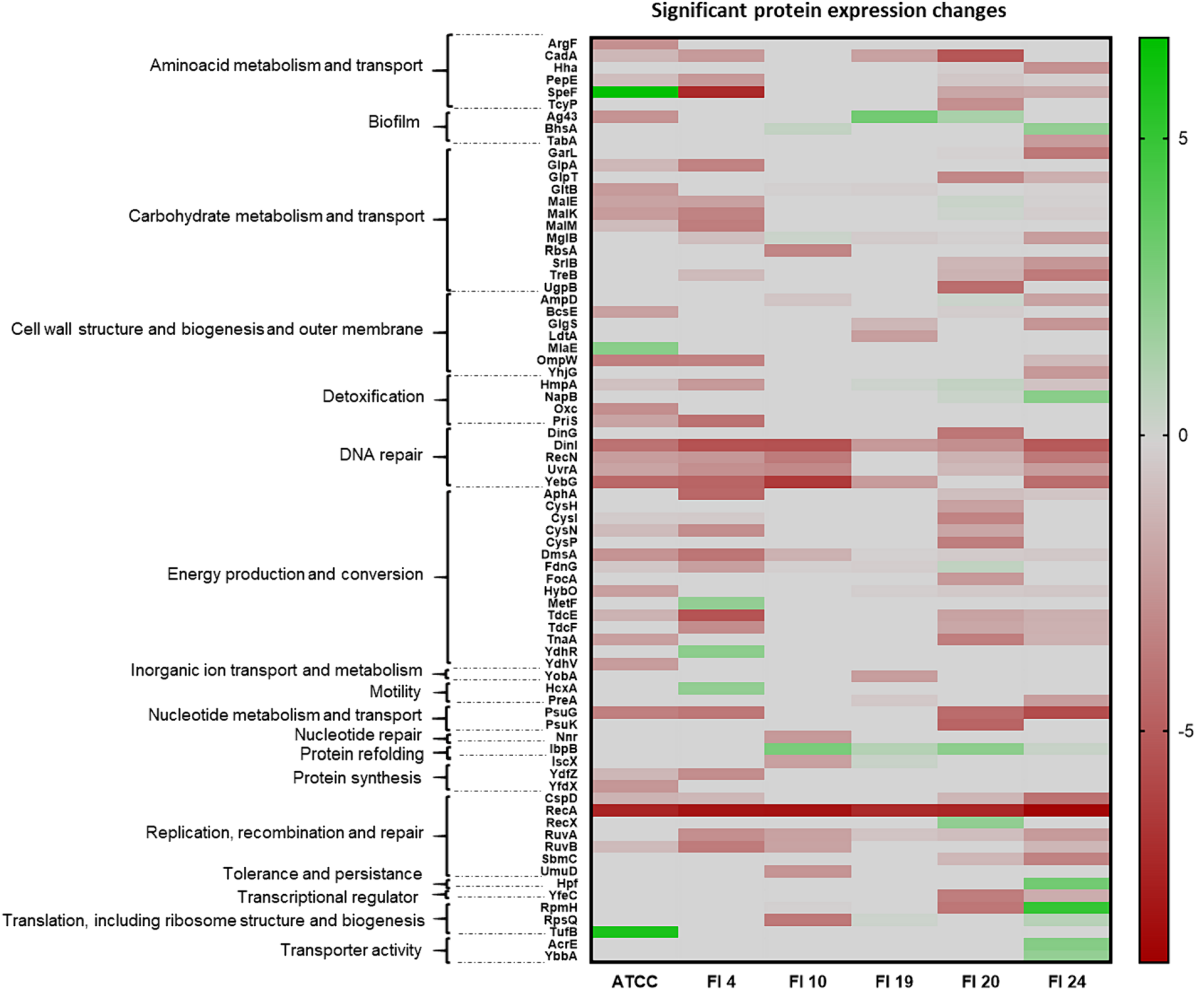
Heatmap of relative protein expression based on label-free quantification by liquid chromatography/mass spectrometry (LC-MS). The main function associated with each protein is shown.

All proteins that showed as significant increase or decrease in their relative expression level upon *recA* deletion (log2 FC >2 or < −2) were analyzed in depth and classified by their function^4^ (Figure 4). Protein expression variations after treatment with ciprofloxacin most affected the following cellular functions: 15/76 (19.7%) proteins were involved in processes of energy production and conversion; 11/76 (14.5%) affected carbohydrate metabolism and transport; 7/76 (9.2%) were involved in replication, recombination and repair, cell wall and outer membrane structure and biogenesis; 6/76 (7.9%) proteins were involved in amino acid metabolism and transport, and 5/76 (6.6%) in DNA repair.

The following cellular processes were most affected and involved changes in expression of different proteins: energy production and conversion (AphA, CysH, CysI, CysN, CysP, DmsA, FdnG, FocA, HybO, MetF, TdcE, TdcF, TnaA, YdhR, YdhV); carbohydrate metabolism and transport (GarL, GlpA, GlpT, GltB, MalE, MalK, MalM, MglB, RbsA, SrlB, TreB, UgpB); replication, recombination and repair (CspD, RecA, RecX, RuvA, RuvB, SbmC, UmuD); cell wall and outer membrane structure and biogenesis (AmpD, BcsE, GlgS, LdtA, MlaE, OmpW, YhjG); amino acid metabolism and transport (ArgF, CadA, Hha, PepE, SpeF, TcyP) and DNA repair (DinG, DinI, RecN, UvrA, YebG; Figure 4).

Once again, the data indicate that the cellular abundance of a large number of proteins decreases under ciprofloxacin pressure in the absence of a functional SOS response.

## Discussion

The SOS response is a conserved bacterial pathway mainly associated with DNA damage repair mechanisms. In this study, using a proteomic approach to study the expression levels of SOS response genes under treatment with a DNA damage-causing agent (ciprofloxacin), we showed significant changes in protein abundance at the cellular level and a correspondingly high variability in protein expression when the clinical isolates were each compared with their isogenic RecA-deficient partner.

The number of genes detected in the collection of selected isolates with different quinolone-resistant phenotypes and the ATCC 25922 reference strain was similar. However, the number of genes shared by each isolate with ATCC 25922 showed high intergenomic variability because clinical isolates were used instead of isogenic strains (as expected, strains ATCC 25922 and FI4, belonging to the same sequence type ST73, conserved the highest percentage of gene identity). High proteomic variability between isolates was also observed when the SOS response was suppressed after treatment with ciprofloxacin. In general, the result of this suppression was a large number of proteins with decreased cellular abundance under ciprofloxacin-induced pressure.

Sequential timing of promoter activation in the SOS response could impact bacterial physiology. Many changes in protein expression levels were probably not detected because exposure to ciprofloxacin in our assays was brief. An striking feature of the LexA/RecA regulatory circuit is that the timing, duration, and the induction level can vary for each LexA-regulated gene, depending on the location and binding affinity of the LexA box relative to the strength of the promoter. As a result, some genes may be partially induced in response to even endogenous levels of DNA damage, while others appear to be induced only when DNA damage to the cell is high or persistent (Courcelle et al., 2001; Culyba et al., 2018).

Despite the genomic and proteomic variability between isolates, the SOS response remained stable and conserved in all of them. In fact, most of the known genes regulated by the SOS system were identified in all isolates. As a result, the impact of suppression of the SOS response in the clinical isolates on sensitization and lethality was similar to that observed in laboratory strains (Recacha et al., 2017; Machuca et al., 2021). In previous studies, the impact of suppression of the SOS response in the presence of ciprofloxacin was analyzed in the clinical isolates that were selected for this study (Machuca et al., 2021). RecA inactivation resulted in 2 to 16-fold reductions in fluoroquinolone MICs, and a change in EUCAST clinical category for FI 4 (LLQR) and FI 20 (LLQR). In addition, a bactericidal effect (a > 3 log_10_ decrease in CFU/mL) was observed after short time intervals (2–8 h) against clinical isolates and their *recA* mutants. After 8 h, no viable bacteria were recovered for FI4 Δ*recA*, FI20 Δ*recA,* and FI24 Δ*recA*. The results clearly showed that suppression of the SOS response in clinical isolates with LLQR, susceptible and resistance phenotypes to quinolones was detrimental to bacterial survival. The data in the present study indicate that, in addition to the proteins involved in the SOS response, the cellular abundance of a large number of proteins generally decreases under ciprofloxacin-induced pressure in the absence of a functional SOS response (Figure 1), and could contribute to an increased susceptibility and a decreased evolution of the *E. coli* isolates toward ciprofloxacin resistance mediated by suppression of the SOS response.

In another previous study by our group, which aimed to better understand the underlying molecular systems responsible for the reduction of bactericidal effect during antimicrobial therapy and to define new antimicrobial targets, the transcriptome profile of isogenic *E. coli* isolates harboring quinolone resistance mechanisms (LLQR phenotype) was evaluated in the presence of a clinically relevant concentration of ciprofloxacin (1 mg/L). In LLQR strains, a marked differential response to ciprofloxacin of either upregulation or downregulation was observed. Multiple genes involved in ROS modulation (related to the TCA cycle, aerobic respiration, and detoxification systems) were upregulated, and components of the SOS system were downregulated (Machuca et al., 2017).

In the present study, the number and type of proteins with significant differential expression in isolates with suppression of the SOS response varied among the isolates. Most of the significant proteins (*p* < 0.05) were underexpressed (Figure 3) and, of these, the most frequently underexpressed in the majority of the isolates (log2 FC < −3) were associated with replication, recombination, and DNA repair processes (DinI, YebG, RecN, UvrA, RuvA, RuvB, CspD; Yamanaka et al., 2001; Kreuzer, 2005); energy production and conversion (AphA, CysI, CysN, DmsA, HybO, TdcE, TdcF, TnaA) and amino acid (CadA, PepE, SpeF) carbohydrate (MglB, TreB) and nucleotide (PsuG) metabolism and transport processes (Karp et al., 2018). Notably, CadA (log2 FC to −5; inducible lysine decarboxylase) plays a role in pH homeostasis by consuming protons and neutralizing the acidic by-products of carbohydrate fermentation (Kanjee and Houry, 2013); DinI (log2 FC to −5; DNA damage-inducible protein I), involved in SOS regulation, inhibits RecA by preventing RecA from binding to ssDNA (Yasuda et al., 1998); YebG (log2 FC to −6; DNA damage-inducible protein) is involved in DNA repair (Lomba et al., 1997); TdcE (log2 FC to −5; 2-ketobutyrate formate-lyase/pyruvate formate-lyase 4) is responsible for transforming pyruvate into fumarate; PsuG (log2 FC to −6; pseudouridine-5′-phosphate glycosidase) is involved in the catabolism of pseudouridine (Preumont et al., 2008). Of note among the overexpressed proteins is IbpB (small heat shock protein), which was upregulated in most strains and associated with misfolded protein repair (Piróg et al., 2021). Other relevant proteins that were overproduced in individual isolates were Ag43 (log2 FC = 3), which favors biofilm formation and fights phage infection (van der Woude and Henderson, 2008); Hpf (log2 FC = 3; ribosome hibernation promoting factor), linked to increased persistence (Song and Wood, 2020); RpmH (log2 FC = 5; 50S ribosomal subunit protein L34), an inhibitor of biosynthetic ornithine and arginine decarboxylases (Panagiotidis and Canellakis, 1984), which are involved in the biosynthesis of polyamines; TufB (log2 FC = 5.8; translation elongation factor Tu 2), where EF-Tu binds aminoacyl tRNAs enabling protein synthesis (Weijland et al., 1992).

Our data indicate that quinolone sensitization as a result of suppression of the SOS response produces changes at the cellular level that involve genes other than those controlled by that stress response system (recombination and DNA repair processes), and it is observed despite the proteomic variability of response in clinical isolates. In other words, our study shows that a coordinated response is needed to enable the cell to combat quinolone-induced genotoxic damage. This involves the significant participation of multiple processes of the central metabolism of the bacteria, among which, in our study, the production and conversion of energy, amino acid, carbohydrate and nucleotide metabolism and transport processes stand out.

The data from our study validate previous studies that used Gram-positive and other Gram-negative bacteria as a model. Using *P. aeruginosa* after treatment with sub-MIC and MIC levels of ciprofloxacin demonstrated the involvement of the SOS response in the downregulation of genes encoding proteins involved in general metabolism and DNA replication/repair, as well as downregulation of genes involved in cell division, motility, quorum sensing, and cell permeability. These changes may contribute to pathogen survival during therapy (Cirz et al., 2006). With respect to *S. aureus*, overall, ciprofloxacin also appeared to induce the downregulation of its metabolism, but with a concomitant increase in TCA cycle activity and error-prone DNA replication. Induction of the TCA cycle appeared to be unique to *S. aureus*. Interestingly, increased utilization of the TCA cycle in this pathogen has been associated with virulence (Cirz et al., 2007). In our study, overexpressed proteins were mainly associated with persistence (Hpf) and biofilm formation (Ag43); however, other proteins were involved in protein synthesis (EF-Tu, TufB, RpmH) and protein refolding (IbpB).

In conclusion, the present study highlights the close relationship between the survival mechanisms of cellular stress response and bacterial metabolism (Lopatkin et al., 2021; York, 2021; Zampieri, 2021). This proteomic approach could contribute to the search for new therapeutic targets against resistant bacteria under genotoxic antimicrobial agents such as quinolones.

## Data availability statement

The datasets presented in this study can be found in online repositories. The names of the repository/repositories and accession number(s) can be found at: https://www.ncbi.nlm.nih.gov/genbank/, PRJNA1015411.

## Author contributions

ER: Writing – original draft, Writing – review & editing. BK: Methodology, Writing – review & editing. SD-D: Writing – review & editing. AG-M: Data curation, Writing – review & editing. EG-T: Data curation, Writing – review & editing. FD-P: Writing – review & editing. AR-R: Supervision, Writing – review & editing. JR-M: Supervision, Visualization, Writing – review & editing.

## Footnotes

^1^ http://www.eucast.org

^2^ https://www.genomicepidemiology.org/services/

^3^ www.graphpad.com

^4^ https://ecocyc.org
